# Geochemical assessment of soil and water around abandoned gold mine tailings dams: evidence from the Welkom Goldfield, South Africa

**DOI:** 10.1007/s10653-026-03513-0

**Published:** 2026-10-04

**Authors:** Rinae Makhadi, Robert Hansen, Kim Dowling, Hassina Mouri

**Affiliations:** 1 Department of Geology, University of the Free State, Bloemfontein, South Africa; 2 STEM College, RMIT University, Melbourne, Australia; 3 Department of Geology, University of Johannesburg, Johannesburg, South Africa

**Keywords:** Abandoned mine tailings, Acid mine drainage, Contamination factor, Ecological risk, Geoaccumulation index, Water quality index

## Abstract

Gold mining in Welkom, South Africa, has generated abandoned mine tailings containing potentially toxic elements, some located near residential and agricultural areas. This study evaluated soil and water geochemistry around abandoned tailings, emphasising ecological and human-health risks. Soil and water samples were collected near and away from abandoned mine tailings and analysed using Inductively Coupled Plasma Mass Spectrometry (ICP-MS) and Inductively Coupled Plasma Optical Emission Spectrometry (ICP-OES). Soil samples were assessed for total and leachable element concentrations. Water contamination was quantified by computing a drinking water quality index (WQI). Mean soil concentrations (mg/kg) were highest for Fe (18,425), Mn (273) and Cr (200), often highest near tailings. Uranium, detected for the first time in the area, reached 49 mg/kg. Manganese had the highest leachable concentration (15 mg/kg), while As ranged from 3 to 304 mg/kg and exceeded the Dutch (2000) and South Africa’s Department of Environmental Affairs (2008) regulatory limits of 29 and 5.8 mg/kg, respectively. Contamination indices indicated low to high contamination of As, Co, Cr, Ni, Pb and U. Considerable potential ecological risks (E_r_^i^), were noted for As (630) and Pb (85). In water, As, Mn and U exceeded the WHO (2022) drinking water and South Africa’s National Water Act (1998) discharge limits indicating contamination, with acidity driving mobilisation of chemical elements. High WQIs revealed that most of the water is unsuitable for drinking. These findings highlight notable mining related impacts on soil and water and further multidisciplinary research on dust, food crops and human exposure pathways are recommended.

**Supplementary Information:**

The online version contains supplementary material available at https://doi.org/10.1007/s10653-026-03513-0.

## Introduction

The gold mining industry has contributed significantly to the global economy, with countries such as China, Australia, Russia, Canada, the United States of America, and Ghana in the forefront of production, with South Africa currently occupying the 12th position (World Gold Council, 2026). The discovery of gold in South Africa’s Witwatersrand Basin in the nineteenth century was one of the most significant mineral discoveries in the world, with approximately 50 200 t of gold extracted and an estimated reserve of 48 100 t (Handley, 2023). Gold mining in the Witwatersrand basin has propelled the country’s economy and established it as one of the most industrialised in Africa (Belle et al., 2021). However, extensive gold mining and related processing in the Witwatersrand goldfields resulted in significant quantities of gold mine tailings distributed across the goldfields (Tibane & Mamba, 2022a). Chetty et al. (2021), estimates that there are about 270 tailings facilities in the Witwatersrand goldfields.

Gold mine tailings contain numerous contaminants; these originating from gold containing ore and the metallurgical extraction processes (Belle et al., 2021). According to Gerstmann et al. (2022), Witwatersrand tailings comprise quartz (70–80%), mica (10%), chlorite and chloritoid (9–18%) and sulphide minerals, dominantly pyrite, and its oxidation product, jarosite (0.5–2 wt%). The oxidation of pyrite in the Witwatersrand tailings serves as a source of sulphuric acid resulting in acidity, increased sulphate and elevated toxic metal concentrations, and the mobilisation of metals (Grover et al., 2016). Toxic metals are also referred to as potentially toxic elements (PTEs) which include a range of metals and metalloids such as, arsenic (As), cobalt (Co), copper (Cu), chromium (Cr), mercury (Hg), nickel (Ni), lead (Pb), uranium (U) and zinc (Zn) (Fashola et al., 2016; Laker, 2023). The PTEs in gold mine tailings are dispersed into the environment by surface water flow, blown away by wind as dust, and rainwater runoff, subsequently reaching surrounding residential areas and agricultural lands (Shabalala et al., 2022; Tibane & Mamba, 2022a). Exposure of human beings to PTE levels exceeding tolerable limits can result in adverse health effects, as most PTEs are not biodegradable and can bioaccumulate in the human body over time (Balali-Mood et al., 2021).

According to Tibane and Mamba (2022a), gold mine tailings disrupted ecosystems in the Witwatersrand basin through soil and water contamination; thus, making it one of the areas with the greatest environmental impact from trace metal contamination from gold mine tailings. Nwaila et al. (2017), noted elevated concentrations (mg/l) of Fe (627), Ni (2.8) and Pb (9.4) in water from Davidsonville, near the old Princess mine gold tailings dam in Johannesburg. Ngole-Jeme and Fantke (2017) reported elevated mean concentrations (mg/kg) of As (1000), Co (1000), Ni (1100) and Zn (5340) in soil from the Witwatersrand basin in Johannesburg. The Welkom goldfield is one of the nine Witwatersrand goldfields and gold mining in the area produced numerous gold mine tailings dams. The Welkom goldfield, like other mining areas, is not exempt from environmental contamination by PTEs from mine tailings. Research on water and soil quality in the area reports elevated and toxic concentrations of Fe and Pb in the groundwater (Belle et al., 2021) as well as the presence of As, Cd, Co, Cu, Cr, Fe, Pb, Se, Ni and Zn in soils form the area, which exceeds the maximum permissible soil quality limits (Belle et al., 2022). Previous work on the distribution of PTEs from mine tailings in the area focused on the concentrations of specific elements (As, Cd, Co, Cr, Cu, Fe, Ni, Pb, Se and Zn) in the soil and groundwater (Belle et al., 2021, 2022). The full range of chemical elements that may be transported in the groundwater and soil as well as the quality of surface water in the vicinity of mine tailings have not been reported. Considering the broad spectrum of chemical elements that can emanate from tailings dams, as shown by other studies (Chetty et al., 2021; Nwaila et al., 2017; Raji & Palamuleni, 2023; Tibane & Mamba, 2022a) in similar geoenvironments, and the ways in which tailings can affect surface water resources, it is important that all the chemical elements in the soil, ground and surface water are investigated.

The aim of this study was to perform a geochemical and ecological risk assessment of PTEs in soil and water from the Welkom gold mining area by: (i) quantifying the broader suite of chemical elements in soil, ground and surface water in the Welkom goldfield; (ii) determining the degree of soil and water contamination by using a combination of contamination indices; contamination factor (CF), geoaccumulation index (I_geo_), pollution load index (PLI), water quality index (WQI) and comparing with regulatory limits; (iii) assessing potential ecological risk (E_r_^i^) and comprehensive potential ecological risk index (PERI) related to the exposure to contaminated soil; (iv) map the spatial distribution PTEs in soil to investigate regional variability. From this multifactor approach, the study contributes to the broader understanding and body of evidence regarding the presence of abandoned mine tailings and dispersal of PTEs in the environment surrounding the mine tailings, which provides a scientific basis for future monitoring and remediation efforts.

### Study area

Welkom is a mining town located in the Lejweleputswa District, within the Matjhabeng Local Municipality in the Free State Province of South Africa. It is located approximately 160 km northeast of Bloemfontein and about 250 km south of Johannesburg, which was established after the discovery of gold in the nineteenth century (Belle et al., 2021). Land use in the area is characterised by commercial mining and manufacturing activities as well as extensive commercial agricultural activities including crop cultivation and livestock farming (Belle et al., 2021; COGTA, 2020). There are also small farming communities in the area that depend on groundwater resources for domestic water supply (Belle et al., 2021; COGTA, 2020). Mine tailings are distributed in different parts of the town, and some are near residential areas and farming lands. Some tailings are completely devoid of vegetation and remain exposed to various agents of erosion, allowing wide spatial dispersion of tailings particles.

**Fig. 1:**
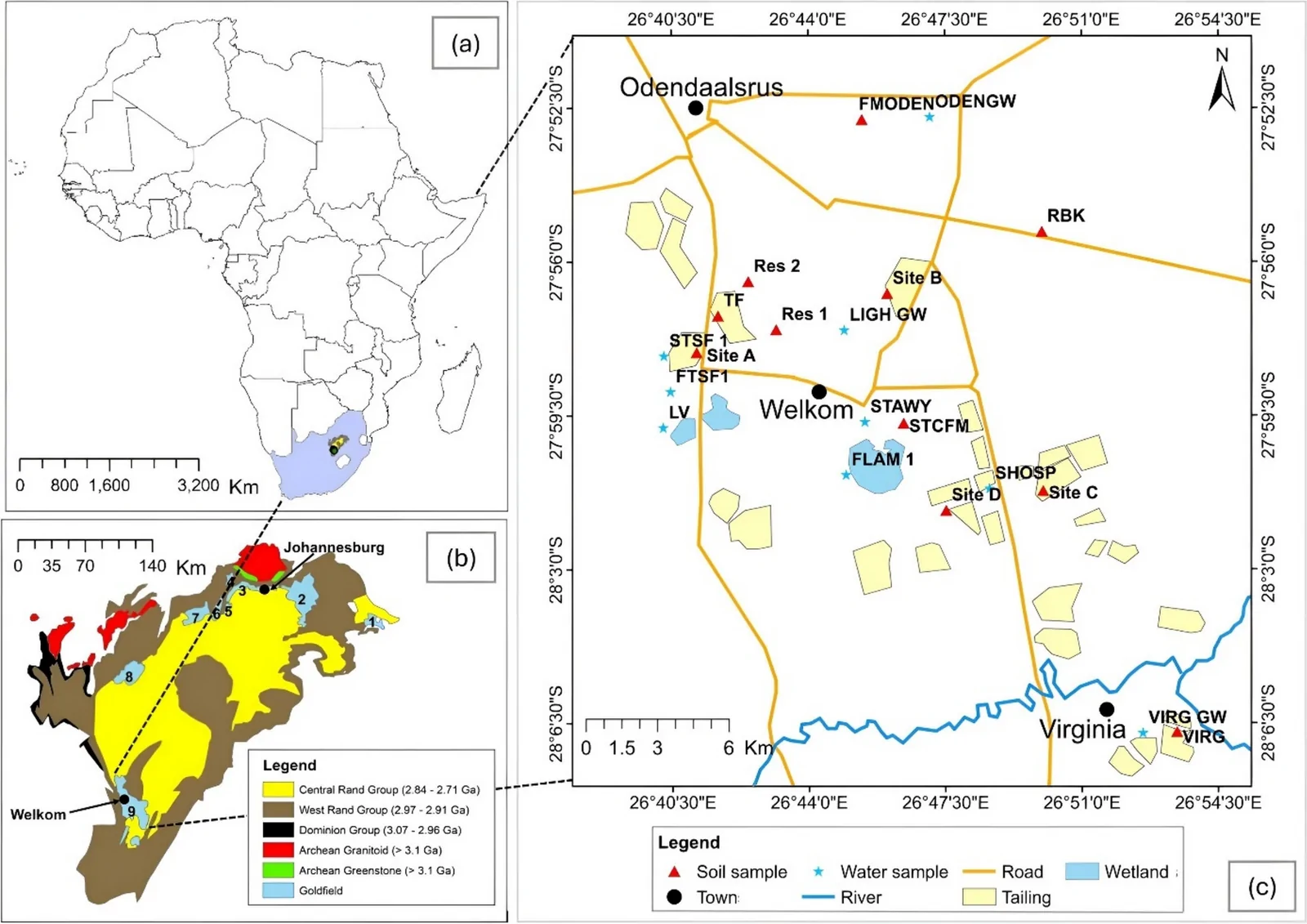
**a** Location of the Witwatersrand basin in the context of Africa and South Africa; **b** geology of the Witwatersrand basin and the respective goldfields; **c** sample location map of the Welkom goldfield and surrounding mining towns

Geologically, the Welkom area is characterised by three units, namely the Witwatersrand Supergroup, Ventersdorp Supergroup and the Karoo Supergroup (Fig. 1b). The Witwatersrand Supergroup overlies ~3.0 Ga granites of the Kaapvaal Craton and the Dominion Group ~ 3.07–2.9 Ga (Philips et al., 2024). The Witwatersrand Supergroup in the Welkom area comprises alluvial quartzites and auriferous conglomerates of the Central Rand Group that host the bulk of gold deposits and are associated with minerals such as pyrophyllite, pyrite and uraninite (Frimmel & James, 2021; Philips et al., 2024). The local hydrogeology includes the fractured aquifers of the Witwatersrand and Ventersdorp Supergroups at varying depth of between 10 and 25 m with yields of between 0.5 and 2 L/S (WRC, 2013).

## Materials and methods

### Soil and water sampling

Spatially representative samples were collected at the locations shown in Fig. 1c. Sites with abandoned mine tailings, tailings footprints and proximity of mine tailings to residential and farming areas were selected. Variations in soil and water were assessed by collecting samples in farming and grazing areas near mine tailings in neighbouring gold mining towns (Odendaalsrus and Virginia).

*Soil sampling*: Sixteen soil samples were collected from eleven sampling locations (Fig. 1c), including two from the vicinity of each of the four abandoned tailings dams (sites A, B, C and D) and the tailings footprint (TF), two from informal residential areas (Res 1 and Res 2) near the tailings footprint and three from farming and grazing lands near Welkom (STCFM) and surrounding mining towns (FMODEN and (VIRG) were selected. Mean concentrations of two samples were calculated to give a composite value for each tailings dam and the tailings footprint. A control sample was collected from an open land area in Riebeeckstad (RBK), which has similar lithology to that of the study sites but with limited human disturbance from mining activities. Soil sampling was conducted according to the sampling guide of the Water Research Commission (WRC) guidelines (Herselman & Moodley, 2009). Soil samples were collected using a stainless-steel handheld auger up to a depth of 30 cm in the topsoil. The soil collection augers were cleaned with distilled water after collecting at each sampling point. All the samples were placed in separate, clearly labelled self-sealing polyethylene bags for the analysis of physicochemical parameters and a full range of total and leachable chemical elements. Soil samples were transported to the soil sciences laboratory facilities at the University of the Free State for preparation and analysis of physicochemical parameters.

*Water sampling:* Four groundwater and five surface water samples were collected to evaluate their physicochemical characteristics and geochemical composition (Fig. 1c). One groundwater sample was collected in an urban residential area near Welkom (LIGH GW). Three groundwater samples were collected from farmlands/grazing land areas where soil samples were collected (ODEN GW, STAWY, VIRG GW). Surface water samples were collected from two wetlands (FLAM 1 and LV) and from surface water (STSF 1, FTSF1 and SHOSP) in the vicinity of site A and C tailings dam (mine water) where soil samples were collected. Water samples were collected according to the sampling procedure outlined by the Department of Water Affairs and Forestry (DWAF, 2000). Water samples were collected directly into high-density polyethylene 1 L plastic bottles for analysis of physicochemical parameters (pH, EC, TDS, DO), anions SO_4_^2−^, HCO_3_^−^, Cl^−^, F^−^, a full range of cations and chemical elements. Prior to collection, bottles were rinsed with sample water to avoid contamination. Sample bottles were labelled with the site name, time and date of collection and were stored in a cooler box containing ice for the sampling duration. Samples were transferred to a fridge after each day’s fieldwork and kept at 4 °C until transportation to the laboratory for analysis.

### Sample pretreatment, laboratory analysis and quality control

*Soil samples:* 1 kg of each soil sample was repackaged into clearly labelled zip-seal polyethylene bags and transported to the WaterLab commercial laboratory for the determination of total and leachable chemical elements concentrations using ICP-MS analysis. 100 g of the soil was prepared for analysis of basic soil parameters, including pH and EC. For the initial pretreatment, samples were dried in an oven at 38 °C for 48 h to remove moisture. Dried samples were ground using a mortar and pestle and sieved through a (≤ 2 mm) sieve to obtain homogenous samples. Sieved soil samples were stored in labelled plastic containers under controlled conditions in the laboratory until further analysis.

For pH, 10 g of sieved soil was transferred into a plastic beaker wherein 25 mL of distilled water was added. The mixture was initially stirred with a glass rod for 5 s, then allowed to settle. It was then stirred again after 50 min and left to stand for a further 10 min. The pH was measured 30 s after the 10-min resting period using a calibrated LASEC FiveEasy F20 benchtop metre electrode. The electrode was rinsed with distilled water in a beaker after each sample was analysed.

For Electrical Conductivity (EC), 20 g of sieved soil was transferred into a 250 mL polyethylene plastic bottle with 100 mL distilled water. The bottles were tightly closed and placed on a shaker for one hour and were allowed to settle for 30 min prior to analysis. The EC was thereafter measured using a calibrated Adwa AD8000 EC meter. The electrode was rinsed with distilled water in a beaker after each sample was analysed.

For chemical elements, analysis included both total and leachable concentrations. For total metal concentrations, microwave digestion using concentrated hydrofluoric and nitric acid (HF-HNO_3_) was employed. 0.5 g of each dried sample was placed in a microwave digestion vessel wherein 9 mL HNO_3_ (65%) and 3 mL of HF (40%) were added onto the sample drop by drop to homogenise the sample. The vessel was sealed and placed in a microwave digestion system (US EPA method, 3052). The solution was cooled to room temperature and then transferred to a flask for analysis. For the leachable concentrations, the ASLP (Australian Standard Leaching Procedure, AS 4439.3–1997) for waste sediments and contaminated soils, part 3: Preparation of leachates was employed. 1 g of each dried sample was digested in 20 mL deionised water. The mixture was placed in a container and mixed for 18–24 h. After the leaching period, the leachate was separated from the solid residue and analysed for the specific chemical elements of interest using ICP-MS (Disfani et al., 2012).

*Water samples*: Water pH, EC and bicarbonate (HCO_3_^−^) measurements were completed with an 855 Robotic Titrosampler. A Hach DO meter (HQ40d) was used to measure dissolved oxygen (DO). Total dissolved solids (TDS) concentrations were determined using a gravimetric method. Concentrations of anions Cl^−^ and SO_4_^2−^ were quantified using a Gallery plus Discrete Analyser. For F^−^, a Hanna Fluoride meter (HI5522-02 ISE Meter) was used. 10 mL of each sample was filtered through a 0.45 µm filter and preserved with concentrated nitric acid for cation and metal analysis. An AVIO 200- ICP OES was used to quantify cation concentrations (Ca^2+^, Na^+^, K^+^, Mg^2+^) and the full range of chemical elements.

To ensure data reliability, rigorous quality assurance and quality control (QA/QC) protocols were implemented throughout all the stages, from sample collection to laboratory analysis of both soil and water samples. Contamination was stringently controlled by subjecting all materials to rinsing with distilled water and air drying, while reagent blanks were used to compensate for background contamination. For water physicochemical analysis, two standard buffer solutions and standard conductivity solutions (1413 µS/cm) were used to calibrate the instrument and meters with automatic temperature compensation were used to ensure accurate readings. Analytical precision was evaluated using duplicate samples, whereas accuracy was determined by using Certified Reference Materials (CRMs) as shown in Tables S1 (soil) and S2 (water). Although the full range of chemical elements were analysed, the tables only include analytical figures of merit and CRMs for the chemical elements that were noted in elevated concentrations and interpreted in the results. The CRM Ultraspec Multi element Aqueous CRM/CRM LOT#Water011-02025us was used for all cations (Table S1b; Table S2b) and NaCl reagent plus ≥99%, Na_2_SO_4_ AM1652849 and CH₃COONa 3H₂O 40387 were used for anions Cl^‑^, SO_4_^2−^ and F^−^, respectively (Table S2d).

The percentage recoveries for all tested chemical elements were within laboratory acceptable ranges of 100 ± 30% for soil and 100 ± 5% for water (Tables S1 and S2). Strong correlation between instrument response and analyte concentration was demonstrated by R^2^ values, which all exceeded 0.99. The limits of detection (LOD) and limits of quantification (LOQ) for each chemical element are listed in Tables S1 and S2. Precision was verified through replicate analyses of some samples and reference materials to minimize errors.

## Data analysis

### Assessment of contamination levels in the soil and potential ecological risks

To assess the degree of soil contamination and quantify potential ecological and human risks, I_geo_, CF, PLI, E_*r*_^*i*^ and PERI for the various PTEs and sampling sites were calculated as described by Saroop and Tamchos (2021), Tibane and Mamba (2022a), Belle et al. (2022) and Gan et al. (2022). These indices are widely used to assess anthropogenic pollution (Yang et al., 2026). Calculations were done using total chemical element concentrations. Table 1 is a summary of the equations, descriptions, and respective categories/classes for each statistical index. To assess compliance of the soil with South African standards, the concentrations of PTEs in the soil were compared with South African soil screening values (SSVs) provided in Table S3. The Dutch target and intervention values for soil remediation (Ministerie van Volkshuisvesting, 2000) were used to assess soil compliance in comparison to international standards. Total chemical element concentrations in soil were compared with leachable fraction of the chemical elements to assess potential dispersion.

**Table 1:** Indices for evaluation of soil contamination and ecological risk, classifications for geoaccumulation index (I_geo_), contamination factor ($\mathrm{C}f$), pollution load index (PLI), ecological risk index (E_r_^i^) and potential ecological risk index (PERI)

Soil pollution indices	Formulae	Description	Classification	References
Geoaccumulation index	I_geo_= Log^2^ $\left(\genfrac{}{}{0pt}{}{Cn}{1.5Bn}\right)$	**Cn**: concentration of element in soil ***Bn***: geochemical background value	**I**_**geo**_**≤0**: practically unpolluted **0<I**_**geo**_**≤1**: unpolluted to moderately polluted **1<I**_**geo**_**≤2**: moderately polluted **2<I**_**geo**_**≤3**: moderately to strongly polluted **3<I**_**geo**_**≤4**: strongly polluted **4<I**_**geo**_**≤5**: strong to very strong pollution **I**_**geo**_**>5**: very strong pollution	Saroop and Tamchos (2021), Belle et al. (2022)
Contamination factor	$\mathrm{C}f= \frac{Ca}{Cref}$	***Ca***: concentration of each metal/ metalloid in the soil sample ***Cref****:* background concentration of the element in the uncontaminated continental crust	***CF* <*1***: low contamination **1 ≤ CF < 3**: moderate contamination **3 ≤ CF < 6**: high contamination **CF≥ 6**: very high contamination	Saroop and Tamchos, (2021), Tibane and Mamba (2022a)
Pollution load index	$PLI=\sqrt[n ]{cf1\times cf2\times ..cfn}$	***n***: number of metals analysed ***CFn***: contamination factors of the different metals	**0–1**: uncontaminated to low levels **1–2**: moderately contaminated **2- 4**: heavily contaminated **4–8**: significantly contaminated **8–16**: extremely contaminated	Tibane and Mamba, (2022a), Saroop and Tamchos, (2021)
Ecological risk index	E_r_^i^ = C^i^_*f*_ ×*T *^*i*^_*r*_	**CF**:*** c***ontamination factor **T**^**i**^_**r**_: toxic response	**E**_**r**_^**i**^** < 40:** low potential ecological risk **40 ≤ E**_**r**_^**i**^** < 80:** moderate potential ecological risk **80 ≤ E**_**r**_^**i**^** < 160:** considerable potential ecological risk	Hakanson (1980) Mohajane and Manjoro (2022)
Potential ecological risk index	PERI = ${\sum}_{i=1}^{n}E$_r_^i^	***n***: number of metals analysed ***E***_***r***_^**i**^: potential ecological risk associated with heavy metal	**<100**: low risk **100–200**: moderate risk **200–400**: high risk **>400**: very high risk	Gan et al. (2022), Liu et al. (2021)

*Geoaccumulation index*: chemical element concentrations in soils were compared to background concentrations in the crust, and a factor of 1.5 was introduced to minimise possible variations in background values for lithological differences (Saroop & Tamchos, 2021). The established background concentrations of selected essential metals in the uncontaminated upper continental crust, as given by Kabata-Pendias and Pendias (2001, in Herselman, 2007), were used (Table S4).

*Contamination factors:* chemical element concentrations in soils were compared with the background or natural concentration in the crust to determine whether a heavy metal was most likely sourced from natural or anthropogenic sources (Saroop & Tamchos, 2021). The concentrations of the selected chemical elements (mg/kg) from the different sampling sites were compared with established background concentrations of essential metals in the uncontaminated upper continental crust, as given by Kabata-Pendias and Pendias (2001, in Herselman, 2007) (Table S4). The contamination factors were used to determine the pollution load index (PLI).

*Pollution load index:* the index was used to assesses the degree of soil contamination (Saroop & Tamchos, 2021) and calculated as the *n*^th^ root of the product of the CF values. Where *n* equalled the number of metals analysed and CF 1, CF2…CF*n* were the CF of the different chemical elements (Saroop & Tamchos, 2021).

*Ecological risk index:* the index measured the potential ecological risk posed by an individual element in soil or sediment. It was calculated as the product of CF for a single element and the T_r_^i^ which was the toxicity coefficient given by Hakanson (1980, in Mohajane & Manjoro, 2022). The toxic response factors of the individual elements were: As (10), Cu (5), Ni (5), Zn (1), Pb (5), Fe (1), Al (1), Mn (1), Ti (1), U (1) and V (1) with no T_r_^i^ values for Ba and Co.

*Comprehensive potential ecological risk index:* the concentration of PTEs combined with their ecological, environmental, and toxicological effects to assess potential risks (Gan et al., 2022). It is the sum of individual risk factors of all the chemical elements analysed in a sample.

#### Comparison of soil PTEs concentration to South African SSVs

Soil compliance with South African standards was assessed by comparing total chemical element concentrations (mg/kg) with South African national norms and standards for the remediation of contaminated land and soil quality, specifically the soil screening values for chemical elements as given by the Department of Environmental Affairs (DEA, 2008) (Table S3). Comparison was done against SSV1 (all land use protective of the water resource) and SSV2 (informal residential areas and commercial/industrial areas). This was done by determining the difference between the measured total chemical element concentration and the allowable SSV value given in Table S3; a positive value indicates contamination, and a negative value indicates uncontaminated soils.

#### Correlation and comparison between total and leachable chemical element concentrations

Using a Spearman’s correlation matrix, correlations were tested between the total and leachable concentrations of chemical elements, which showed potential contamination with respect to the SSVs. A positive value represented a positive monotonic value, and a negative value indicated a negative monotonic relationship; the closer the value was to 1 or − 1, the stronger the relationship (r = 0.87–0.91, *p* < 0.01) (Nyirawasa et al., 2024). Line plots were used to compare total metal concentrations to the leachable fraction of the chemical elements in the soil.

### Assessment of contamination levels in water

To assess water compliance standards, mine water was compared to values given in the South African National Water Act (Act 36 of 1998) for the discharge of industrial wastewater into a resource. For groundwater and wetlands, physicochemical properties and concentration of chemical elements (geochemical composition) were compared to drinking water standards set out by the WHO (2022). To assess the suitability of groundwater and wetlands for drinking and potential anthropogenic water contamination, the WQI was calculated as described by Rakotondrabe et al. (2017). Table S5 summarises the equation and respective categories/classes. The WQI is a commonly used method for describing water quality, which incorporates physical, chemical, and biological parameters into a single value ranging from 0 to 100 (Rakotondrabe et al., 2017). The method involved assigning weights to each parameter from a scale of 1–5, with 1 indicating the least effect on water quality and 5 indicating the highest effect on water quality. This was based on known effects on human health and their overall importance to drinking water quality (Rakotondrabe et al., 2017). Table S6 summarises the parameters weighted for this study and the respective weights assigned to each parameter.

### Assessment of water chemistry and dominant geochemical processes

Piper trilinear plots and Gibbs diagrams were generated to illustrate overall water chemistry and investigate the major geochemical processes controlling it. Piper diagrams depicted the composition of cations and anions in water to understand the main hydrogeochemical types controlled by cations and anions (Chai et al., 2020). This provided insight into the composition of water based on its cation and anion content, which, in turn, determined the source of the water, potential mixing of end members, and hydrogeochemical processes controlling water chemistry (Shelton et al., 2018). Gibbs plots aided depicting the hydrochemistry (rock-water interactions, evaporation and precipitation dominance) of water and in determining the major processes controlling its chemistry (Zhang et al., 2018). The plot illustrated the composition of cations and anions as a function of total dissolved solids (TDS) (Zhang et al., 2018).

### Geospatial, geochemical and statistical analysis

The Garmin (etrex 10) GPS was used to obtain coordinates (latitudes, longitudes and elevations) for the water and soil sampling locations. The data (coordinates) were imported into Google Earth Pro and ArcGIS Pro 3.0.3 software’s to create locality and geospatial distribution maps. For the statistical analysis of the data, which included descriptive statistics and the construction of statistical and geochemical plots, Spearman’s correlation, IBM SPSS Version 30.00, Microsoft Excel, and Python Version 3.10 were used. Piper diagrams were plotted using AquaChem water quality software Version 1.5, to depict water chemistry. Gibbs plots were generated using ArcGIS, Microsoft Word and Excel.

## Results and discussion

### Soil physicochemical parameters and geochemical composition

The statistical summary of physicochemical parameters and the geochemical composition of soil, with comparisons to different permissible limits, is shown in Table 2, while the detailed site-specific concentrations are provided in Table S7. The analysis of soil physicochemical parameters showed variability in pH and EC, with pH values ranging from 3.7 to 7.2 and EC from 28 to 8700 µS/cm. Ten samples revealed acidity, specifically at sites near mine tailings (sites A, B, C, D, TF and VIRG) with site A, TF and VIRG recording the most acidity. This is consistent with findings by Belle et al. (2024), who reported acidic soils in the Witwatersrand goldfields with average pH values ranging between 4.5 and 5.5. The dominant chemical elements in the soil included Al, As, Ba, Cr, Co, Cu, Fe, Mn, Ni, Pb, Ti, U, V and Zn (Table 2; Fig. S1), with Al, Fe and Ti revealing elevated concentrations. These concentrations were evaluated against the Dutch target and intervention values for soil remediation (Ministerie van Volkshuisvesting, 2000) and South Africa’s Department of Environmental Affairs (DEA, 2008) permissible limits. Assessment of compliance levels with these limits showed that As, Cu, Co, Mn, Ni, Pb, U, V and Zn generally adhered to the regulatory limits. Exceptions were noted for As, Cu, Mn, Ni and Pb at some sites. Four sites had high concentrations of As (sites C, D, TF, VIRG), with VIRG revealing the highest concentration of 304 mg/kg. The As concentrations exceeded the Dutch target values of 29 mg/kg and DEA, SSVs limits of 29, 5.8, 23 and 150 mg/kg respectively. Four sites had high concentrations of Cu (sites B, D, TF, RBK, VIRG), two for Mn (sites B, D), two for Ni (sites C, VIRG) and three for Pb (site A, TF, VIRG) where the PTEs exceeded both the Dutch limits and the DEA soil standards for various land use purposes. Cr exhibited 100% non-compliance across all sites, which highlights the severity of Cr contamination in the study area, with site TF recording the highest concentration of 467 mg/kg. U showed the highest concentration of 49 mg/kg at site VIRG; there are no official limits available for U. The strong acidity at sites VIRG and TF coincides with elevated concentrations of As, Cu, Cr, Ni, Pb and U, confirming the relationship between acidic soils and increased metal solubility (Sintorini et al., 2021). A recent study by Belle et al. (2024) noted As, Cd, Cr, Cu, Fe, Ni, Pb, Se and Zn as some of the dominant elements in Welkom goldfield soil which is similar for the current study. The identification of Al, Ti, Mn, Ba, V and U which are elements not previously reported in the area, expands the geochemical profile of the Welkom goldfield to the broader suite of mine tailings contaminated soil. Similar studies in the Witwatersrand basin (Ngole-Jeme & Fantke, 2017; Tibane & Mamba, 2022a) identified As, Co, Cu, Cr, Fe, Mn, Ni, and Zn as common PTEs, with As being the most prevalent. This reveals similarities with regards to the PTEs responsible for soil contamination across the Witwatersrand basin.

**Table 2:** Statistical summary of soil physicochemical parameters (pH and EC) and concentrations of PTEs (mg/kg) in soil collected in the vicinity of abandoned mine tailings in comparison to the respective limits (EC, µS/cm)

	Median (Min–Max)	Mean ± SD	Dutch target values^a^	DEA, SSV1^b^	DEA, SSV2 (informal residential)^c^	DEA, SSV2 (commercial/industrial)^d^	Above limit
pH	5.5 (3.7–7.2)	5.6±1.2	–	–	–	–	–
EC	58 (28–8700)	599±2160	–	–	–	–	–
Al	16,200 (6400–36,000)	18,037±9381	–	–	–	–	–
As	6 (3–304)	35±76	29	5.8	23	150	2^a^, 8^b^, 2^c^, 1^d^
Ba	17 (1–262)	63±78	–	–	–	–	–
Co	10 (10–38)	15±9	–	300	300	5000	0^b^, 0^c^, 0^d^
Cr	156.5 (110–467)	200±112	100	6.5	6	40	16^a, b, c, d^
Cu	18.5 (7–91)	22±20	36	16	1100	1900	1^a^, 5^b^, 0^c, d^
Fe	19,600 (9600–34,000)	18,425±6939	–	–	–	–	–
Mn	175 (70–1112)	273±288	–	740	740	12,000	2^b, c^, 0^d^
Ni	12 (10–64)	20±16	35	91	620	10,000	2^a^, 0^b, c, d^
Pb	14.5 (7–289)	44±72	85	20	110	1900	2^a^, 3^b^, 1^c^, 0^d^
Ti	2674 (1674–24,500)	3918±5511	–	–	–	–	–
U	2.1 (0.8–49)	11±14	–	–	–	–	–
V	57 (43–102)	61±16	–	150	150	2600	0^b, c, d^
Zn	32 (12–105)	44±32	140	240	9200	15,000	0^a, b, c, d^

### Spatial distribution of PTEs in soil

**Fig. 2:**
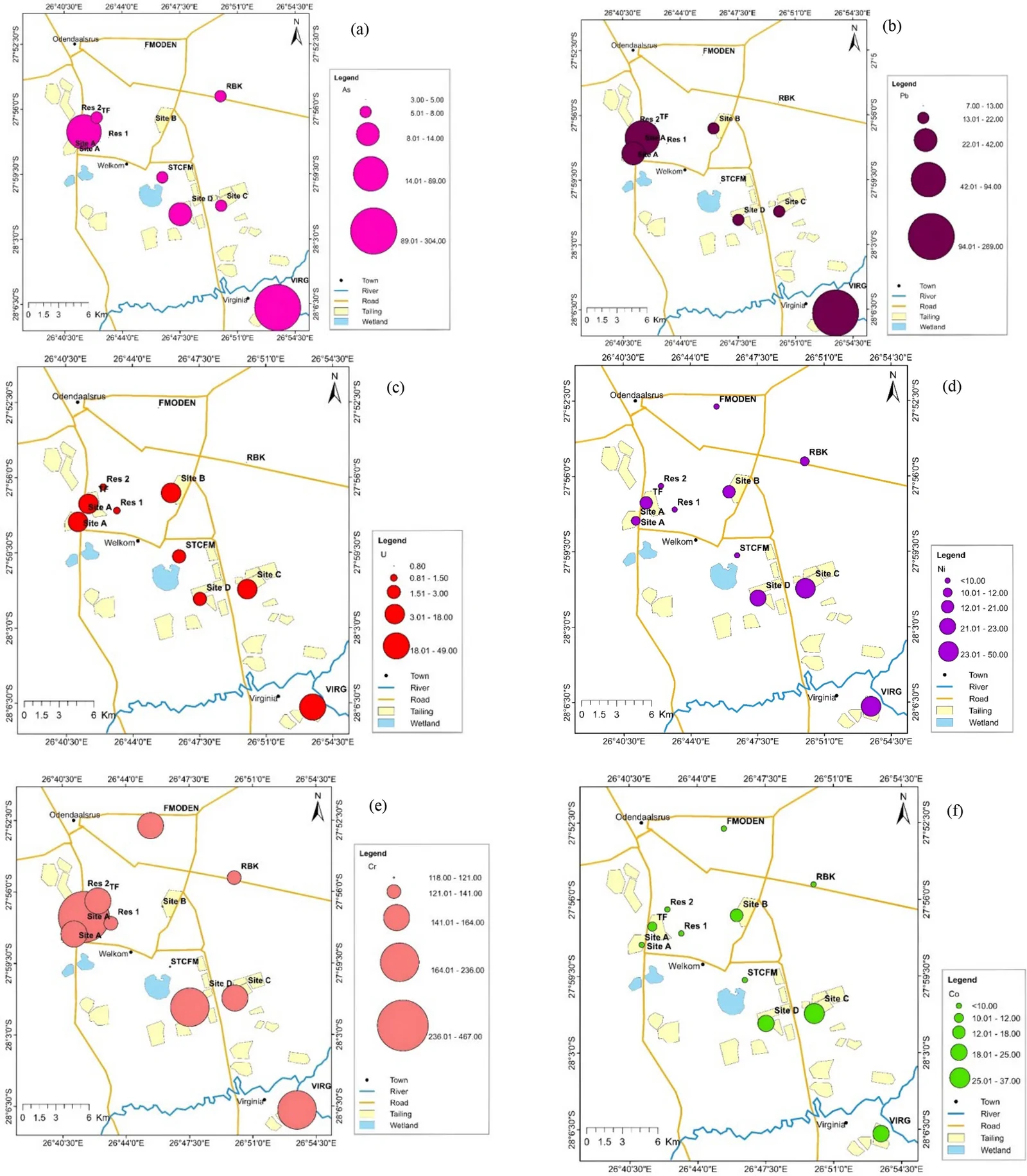
Spatial variations of a) As, b) Pb, c) U, d) Ni, e) Cr, and f) Co in the study area

The spatial distribution maps provided in Fig. 2(a–f) illustrate the distribution of chemical elements across the sampling locations. The maps were constructed for PTEs (As, Cr, Pb, Co, Ni and U) with elevated concentrations and exceeding regulatory limits. Site VIRG portrayed the highest concentrations, particularly for As, Pb, U and Ni (Fig. 2a–d). The maps revealed high concentrations of Cr, mainly concentrated in site TF (Fig. 2e), with Co concentrated in site C, D and Virg (Fig. 2f). The spatial distribution maps reveal that the sampling sites at a distance from the mine tailings such as RBK and FMODEN recorded lower concentrations of PTEs in comparison to those in proximity to the mine tailings, with VIRG representing the most contamination. This provides evidence that mine tailings contribute significantly to concentrations of PTEs in soils.

### Soil contamination and ecological risk

#### Geoaccumulation index, contamination factor and pollution load index

The I_geo_ and CF estimates of the chemical elements are provided in Table S8 and Fig. S2 and S3. I_geo_ values of 0 indicated that all the sites revealed background concentrations/ practically unpolluted for Al, Ba, Mn, Fe, Co, Cu, Ni, Ti, V and this can be attributed to their natural abundance (Kabata-Pendias & Pendias, 2001, in Herselman, 2007). Contrarily, elevated I_geo_ values for As, Cr, Pb and U at various sites indicate anthropogenic pollution. Notably, site TF showed moderate As pollution with an I_geo_ of 2, while VIRG portrayed extreme As enrichment with an I_geo_ of 12. Pb and U ranged from unpolluted to strongly polluted across the sites, with VIRG standing out as the most impacted site with an I_geo_ of 3 (Pb) and 3.6 (U) respectively. These findings are consistent with previous studies in other gold mining regions, such as Akoto et al. (2023), who reported elevated I_geo_ values for As, Cd and Pb due to mine tailings. The CF analyses are coherent with the I_geo_ results with As, Pb and U showing moderate to very high contamination across the sites. Notably, VIRG revealed the highest CF values for As (63), Pb (17) and U (18). Sites A, C, D and TF also portrayed elevated CF values, specifically for Cr, Pb and U. These findings are consistent with findings by Ngole-Jeme and Fantke (2017), who reported high CF values for As, Cd, Co and Ni around gold mine tailings in the Witwatersrand basin, near Johannesburg highlighting the potential risks to human health and the ecosystems. Pollution load index values across the sites ranged from uncontaminated (0–1) to moderately contaminated (1–2), indicating variability across the sites (Table S9). Sites A, Res1, RBK, FMODEN, and STCFM showed uncontaminated, with Sites B, C, D, TF, and VIRG revealing moderate contamination levels and VIRG recording the highest PLI. The high PLI values in VIRG reflect elevated concentrations of various PTEs in soil and how they collectively influence the overall quality of the soil.

#### Ecological risk

Ecological risk index (*E*_*r*_^*i*^) values (Fig. S4) varied across the sites with Al, Cr, Cu, Fe, Mn, Ni, Ti, U and Zn indicating low ecological risk. As and Pb revealed notable *E*_*r*_^*i*^ values, specifically for site TF and VIRG. As reached values of 100 in TF and 630 in VIRG, while Pb recorded 85 in VIRG. Notable *E*_*r*_^*i*^ values were reported by Tibane and Mamba (2022a) who observed an *E*_*r*_^*i*^ of 320 for Cu in gold tailings contaminated soil in the Witwatersrand basin, Johannesburg, concluding that residents were at risk and suggested restrictions on housing developments near the contaminated sites. Although Res 1 and Res 2 in the present study show low ecological risk, their proximity to site TF raises concern, specifically for children, with Kwong et al. (2021) confirming soil ingestion and dust exposures as primary pathways for children’s contact with contaminants in the soil. The comprehensive potential ecological risk (Table S9) supports the above findings with values ranging from very low (14) to high (765), with site VIRG exhibiting the highest risk, followed by TF. de Souza Neto et al. (2020) demonstrated the extent of metal contamination in gold mining regions, reporting values between 10 and 26,524 in the eastern Amazon, indicative of a very high ecological risk. This is also evident in this study as the *E*_*r*_^*i*^ values have revealed the elements that represent significant risk in the study area.

### Comparison of PTEs in soil to South African SSVs

The allowable background SSV value for each element was compared to the measured chemical element concentration as a guide to assess soil contamination (Table S10). Comparisons varied across the sites with Co, Ni, V and Zn returning negative values, indicating no contamination for all sites. Similar findings were reported by Tibane and Mamba (2022b), who reported negative values for Co, Ni and Zn in soils in the Witwatersrand basin, near Johannesburg mine tailings. Contrarily, Cr showed positive values across all sites, confirming contamination with respect to both SSV1and SSV2, with Cu contamination limited to site D, RBK and VIRG. Tibane and Mamba (2022b) reported negative Cr values but positive Cu values for some sites, highlighting variability in contaminants present in the Witwatersrand basin soil. Sites A, D, TF and VIRG demonstrated contamination with Pb, As, Cu and Cr with VIRG consistently emerging as the most contaminated site for both SSV1 and SSV2 and this is consistent with the statistical indices, which identified VIRG as the most impacted site. The absence of regulatory/permissible limits for Uranium in South African soil screening standards is concerning, given the country’s significant uranium reserves (Issah et al., 2025). Gold mining in the Witwatersrand basin contributed to elevated U concentrations, and the accumulated tailings remain as active sources of Uranium that can potentially disperse into surrounding environments (Gerstman et al., 2022). Elevated U levels pose environmental and health risks, including carcinogenic and renal effects (Faqir et al., 2025; Zupunski et al., 2023). These findings highlight the need to include uranium into South African SSVs to ensure compliance monitoring.

### Relationship between total and leachable chemical element concentrations

Strong correlations between total and leachable concentrations (Table S11) of As (r=0.54), Co (r=0.57), Ni (r=0.53), U (r=0.815) and Zn (r=0.675) (r=0.87–0.91, p<0.01) highlight the close relationship between the chemical elements in the soil and the fraction that is potentially mobile and bioavailable. Although the correlations reveal a strong relationship between the total and leachable chemical element concentrations, the actual/ measured leachable concentrations for most elements (As, Ba, Cr, Fe, Pb) remained consistently low across all the sites, suggesting limited bioavailability (Fig. S5a–e). In contrast, Co, Cu, Mn, Ni, U and Zn (Fig. S5f–j) exhibited low but notable leachable fractions, particularly at TF and site C. Similar findings were noted by Jelić et al. (2025), who observed low leachable concentrations despite elevated total concentrations from urban soils in Novi Sad, Serbia. The study further concluded that ecological and health risks estimated from leachable concentrations were lower than predicted from the total concentrations. The estimated pollution and ecological risk indices reported in this study would also be much lower if they were estimated using the leachable concentrations, considering the reported low leachable concentrations in this study.

### Water physicochemical parameters and geochemical composition

The statistical summary of physicochemical parameters and geochemical compositions (mg/L) of water samples are shown in Table S12. Groundwater pH values ranged from 7.2 to 7.9 and were within the WHO (2022) limits, consistent with findings from Belle et al. (2021), who reported pH values within the specified limits in the Welkom goldfield. Electrical conductivity (EC) ranged from 88 to 295mS/m and TDS from 564 to 2092 mg/L with both frequently exceeding the permissible limits and the borehole LIGH GW revealing the highest EC (295 mS/m) and TDS (2092 mg/l), confirming the relationship between EC and TDS (DWS, 1996). The highest concentrations of major ionic species (HCO_3_^−^, SO_4_^2−^, Cl^−^, Ca, Na and K) were also elevated in LIGH GW exceeding the limits and this is coherent with the elevated TDS for the groundwater, with ODENGW revealing the lowest concentrations. PTEs analyses revealed elevated concentrations (mg/L) of Li (0.2–0.12), Mn (0.03–7.4) and Si (6–17), with LIGH GW exceeding the Li permissible limit of 0.06 mg/l and VIRG GW exceeding the Mn limit of 0.08 mg/l. Other PTEs, including; Al, Co, F, Fe, U and Zn were detected but within the WHO (2022) limits. Dorleku et al. (2018) similarly reported elevated concentrations of Mn, Fe, Al and Cu in groundwater from a gold mining region in Ghana and linked these to anthropogenic sources. Elevated PTEs in the groundwater is concerning as the water is used domestically in the area.

The wetlands pH values were within the WHO limits (7.5–9.1) and similarly to groundwater, the wetlands EC (160–1276) and TDS (948–8884) exceeded the WHO limits, and the LV wetland exceeded the limits portraying extreme salinity and elevated concentrations of major ionic species and PTEs including; As, F^−^ and Ni. A study by CMBG (2022) on the Welkom wetlands also noted As concentrations exceeding the WHO limits for drinking water and highlighted potential effects as the communities are dependent on these wetlands for domestic purposes. According to Chetty et al. (2021), the Klip River wetlands in the Witwatersrand basin in Johannesburg receive run-off from the Central rand goldfields with high concentrations of Co, Ni, U and Zn which are naturally sequestered through precipitation and adsorption resulting in downstream remediation of water. The presence of notable concentrations of PTEs in Welkom wetlands provides a platform for further investigation into the various biogeochemical processes in the wetlands to assess their potential to counteract contaminants.

Mine water pH ranged from 2.5 to 7.7, depicting acidic to neutral conditions, with site C (SHOSP) tailings dam showing the most acidity and exceeding the National Water Act (NWA) (No. 36 of 1998) limits (Republic of South Africa, 1998). Electrical conductivity (EC) ranged from 327 to 1102 mS/m and TDS from 4058 to 9352 mg/L, with site A (FTSF1) revealing the highest EC and TDS. According to Laker (2023), extremely low pH between 3 and 4 are common in the waters around mine tailings in the Witwatersrand goldfields and this is driven by Acid Mine Drainage (AMD) that arises from the oxidation of the high levels of pyrite in the mine tailings that produces sulphate that dissolves in the water and produces acid. Major ionic concentrations revealed elevated SO_4_^2−^ concentrations (2403–3698) with site C revealing highest concentration. PTEs concentrations varied with As reaching a maximum concentration of 2 mg/L with SHOSP revealing the highest concentration exceeding the NWA limit of 0.01 mg/L. Apart from As, Fe (2.8–427), Mn (4.3−42), Ni (0.6–13) and U (0.9–5.8) were also present in elevated concentrations, with SHOSP revealing most exceedances. Laker (2023) emphasises that the low pH water in the vicinity of mine tailings enhances the mobilities of metallic PTEs. Similarly, Sako et al. (2018) reported elevated concentrations of As, Cu, Cd, Fe and Ni in surface water from a gold mining region in Tongon, northern Côte d'Ivoire, West Africa and attributed the PTEs to the oxidation and dissolution of the sulphide mineralisation in the area. Although the mine waters are not used domestically, they pose environmental risks due to seepage. Wolkersdorfer and Mugova (2022), emphasises that seepage from mine tailings and rock waste dumps reaches the surface water through direct seepage or groundwater recharge in areas with shallow water resulting in the contamination of water. The interconnectedness of the different water resources promotes the distribution of PTEs from mine tailings in the Welkom goldfield to the surrounding surface and ground water resources.

### Geochemical processes controlling water quality

#### Hydrogeochemical facies

**Fig. 3:**
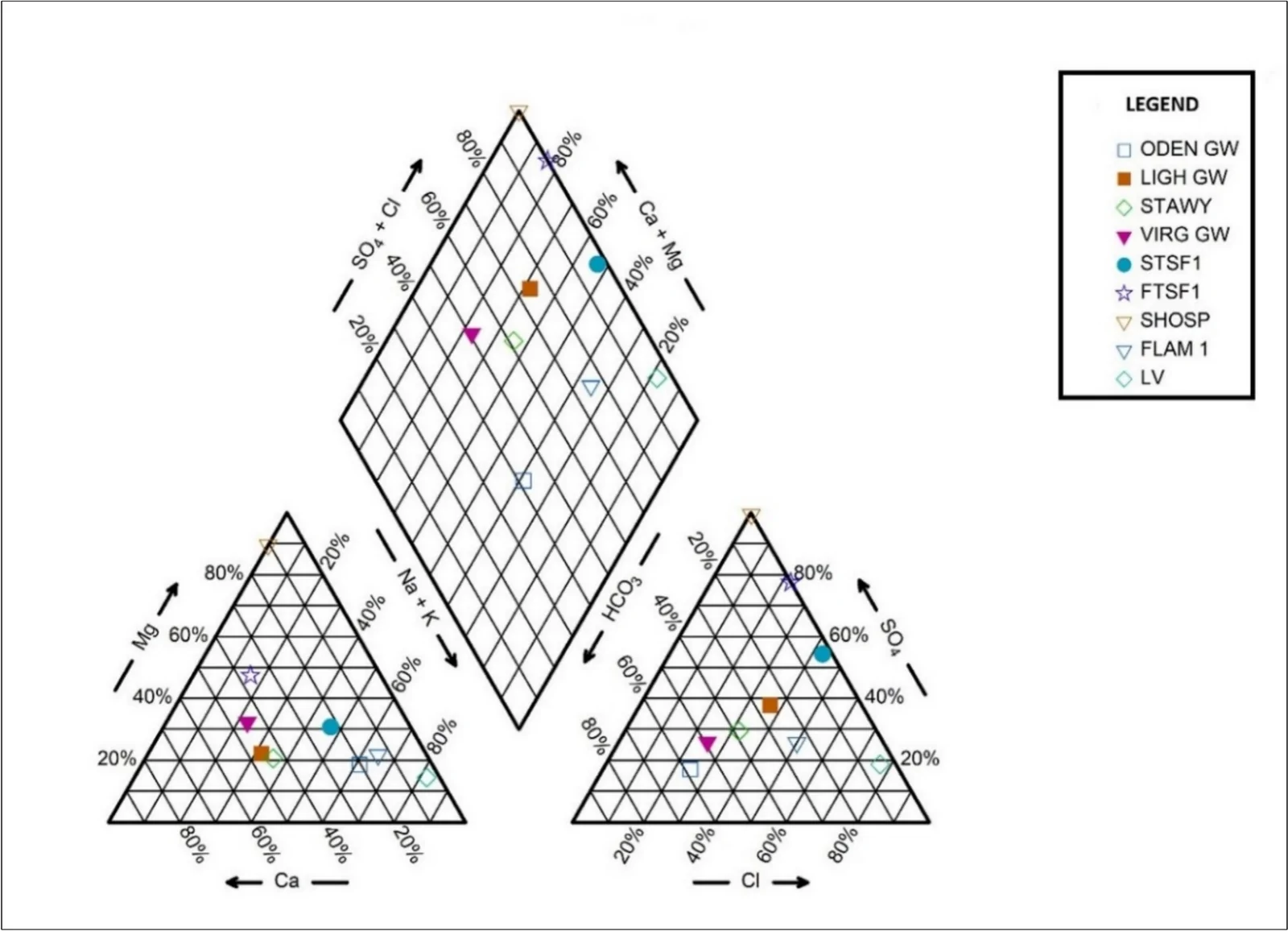
Piper trilinear plot illustrating the major hydrochemical facies for the water samples

The piper’s trilinear diagram (Fig. 3) revealed three hydrogeochemical facies. The two wetlands (sites LV, FLAM 1) belonged to the sodium-chloride (Na^+^- Cl^−^) water types. One groundwater (site ODEN GW) belonged to the sodium bicarbonate (Na^+^- HCO_3_^−^) water types. The (Na^+^- HCO_3_^−^) water type represents silicate weathering and evaporated water with (Na^+^-Cl^−^) reflecting stagnant water or coastal wetlands (Jia et al., 2020). According to Chae et al. (2006), Na^+^-HCO_3_^−^ groundwater represents fresh meteoric groundwater that interacts with silicate rocks or undergoes cation exchange where sodium in sediments is exchanged for calcium in groundwater. The mine waters (sites STSF1, FTSF1, SHOSP) and three groundwater samples (sites LIGH GW, VIRG GW and STAWY) belonged to the calcium magnesium sulphate (Ca^2+^-Mg^2+^- SO_4_^2−^) water types which is common for groundwater and mining impacted waters. According to Erdogan et al. (2020), the Ca^2+^-Mg^2+^- SO_4_^2−^ water type is a key indicator of sulphide oxidation and dissolution in acid mine environments as well as subsequent neutralisation by carbonate minerals and is characterised by high salinity and TDS concentrations due to mineral dissolution.

#### Geochemical processes controlling water quality

The Gibbs classification scheme (Fig. S6) demonstrates that the major geochemical processes controlling water quality include rock weathering and evaporation dominance. Three groundwater samples and one wetland belonged to the rock dominance category with a 50:50 ratio of cations and anions. The rock dominance process entails that the underlying rocks undergo extreme weathering enabling mineral dissolution, ion exchange and mixing between groundwater and aquifer materials (Anjali et al., 2025). The mine waters, one wetland and groundwater sample belonged to the evaporation-crystallisation dominance category. For the mine waters, the anions dominated the overall TDS with 50:50 ratio between cations and anions for the wetlands. Water in the evaporation dominance category are characterised by high TDS concentrations, and this is indicative of increased residence times and intense evaporation as well as pollution (Li et al., 2025). This is coherent with the findings from this study because all the mine waters and one wetland belonged to the evaporation crystallisation dominance category and are all characterised by high TDS concentrations.

### Water contamination

The WQI values in Fig. S7 show that three of the four groundwater samples (sites VIRG GW, STAWY, LIGH GW) had poor quality, revealing water unsuitable for drinking purposes. The two wetlands (site LV, FLAM) varied in terms of the overall water quality, with FLAM indicating good water quality and LV indicating very poor quality. The poor WQI reflects the presence of various PTEs exceeding the limits resulting in the water being unsuitable for drinking purposes. The borehole in the Odendaalsrus farm (site ODENGW) returned good water quality which attributed to the distance of the farm from the surrounding mine tailings. This WQIs are consistent with the overall composition of PTEs and the hydrogeochemical facies as it is the only groundwater that plotted in the sodium bicarbonate water type. The poor quality of the three groundwater samples (sites VIRG GW, STAWY, LIGH GW) and the lakeview wetland (site LV) have the potential to affect surrounding communities negatively as these water resources are used for domestic purposes (Belle et al., 2021; CMBG, 2022).

## Conclusion

This study investigated the geochemistry of soil and water in the vicinity of abandoned mine tailings in the Welkom goldfield, emphasising on ecological and human-health risks. Results show evidence that soil and water resources in the Welkom goldfield are notably impacted by abandoned mine tailings. Soil physicochemical analyses revealed acidic soils near tailings with elevated concentrations of PTEs including; As, Cr, Ni, Pb and U. These findings validate the strong link between soil acidity and increased metal solubility. Site VIRG emerged as the most contaminated, with elevated concentrations of As, Pb and U. Spatial distribution maps revealed that sites near mine tailings and tailings footprints had the highest concentrations of PTEs, highlighting their role as sources of PTEs. Soil contamination and ecological risk indices identified sites VIRG and TF as the most polluted also accompanied by notable ecological risks for As and Pb. The detection of Ba, U and V supports the need for the quantification of all PTEs present in mine tailings contaminated soil. The lack of regulatory limits for U in South Africa reveals the need for the inclusion of U to ensure compliance. Water analyses revealed variations in pH, EC, TDS and PTE concentrations in groundwater, wetlands and mine water. Water quality indices for groundwater and wetlands revealed water unsuitable for drinking, confirming impact of various physicochemical parameters on water quality. The mine water showed the most acidity and diverse concentrations of PTEs reflecting the role of acid mine drainage on the dissolution of PTEs in water.

These findings highlight that abandoned mine tailings are a notable source of PTEs that pose risks to soil and water quality and further potential impacts on human health.

## Recommendations and limitations

To mitigate the risk of PTE contamination, it is recommended that communities in the vicinity of mine tailings should be made aware and use local soil with caution, for example, avoid growing foods in the soils or add topsoil, limit children’s playing around the tailings, ensure hand washing at the end of play and promote the use of dust masks during windy seasons. Residents may be informed of positive ways they can contribute to their wellbeing with education and distribution of information, similar to campaigns in New Zealand (Te Whatu Ora – Health New Zealand, 2023). The farmers in the Virginia grazing land should be informed about the potential risks to families and animals with regards to the proximity of the grazing land to mine tailings. With reference to water quality, this study recommends that the groundwater in some of the areas should be used with caution as there are numerous PTEs that are present in the water, which have the potential to cause long-term undesirable health issues as revealed by WQIs. The physicochemical properties of the mine waters are evidence that mine tailings dams generate acidity, which is linked to the mobilization of various PTEs in the immediate environment and there needs to be a comprehensive solution to prevent the distribution of water from the mine tailings into the surrounding surface and groundwater bodies.

While this study provides valuable insight and a global contribution to understanding the dispersal of PTEs from mine tailings into the surrounding soil and water resources, and the potential ecological and human risks, the analysis of other media such as air-dust and food crops was not performed. This limitation may restrict the detailed understanding of the full extent of the dispersal of PTEs from the mine tailings into the surrounding environment. Ultimately, this study supports the design of a further detailed geochemical assessment of air-dust and food crops in the area as well as the grass in the grazing land. Furthermore, possible human health exposure pathways should also be ascertained and detailed human health investigations should be performed through the distribution of questionnaires to the local communities to identify any health issues that might be prevalent in the area due to exposure to the different PTEs identified in this study.

## Supplementary Information

Below is the link to the electronic supplementary material.

Supplementary file1 (DOCX 1061 KB)

Supplementary file2 (DOCX 77 KB)

## Data Availability

No datasets were generated or analysed during the current study.
