# Supplementary material for: Geochemical assessment of soil and water around abandoned gold mine tailings dams: evidence from the Welkom Goldfield, South Africa
Source: Environ Geochem Health. 2026 Oct 4;48(15):618. doi: 10.1007/s10653-026-03513-0 (PMC13633954; doi:10.1007/s10653-026-03513-0)
Supplement: Supplementary file 1 [file 10653_2026_3513_MOESM1_ESM.docx]

**Fig. S1:** Mean concentrations of PTEs in the soils

**Fig. S2** Geoaccumulation index of PTEs in the soils across the sampling sites

**Fig. S3** Contamination Factors of PTEs in the soils across the sampling sites

**Fig. S4** Ecological risk index (*E_r_^i^* ) for individual chemical elements across the sampling sites

|   (a) |   (b) |
| --- | --- |
|  |  |
|   (c) |   (d) |
|  |  |
|  |  |
|  |  |

(f)

(e)

(b)

|  |  |
| --- | --- |
| (g)   |   (h) |

(j)

(i)

|   (k) |  |
| --- | --- |

**Fig. S5** Comparison between total and leachable concentrations of PTEs in the soil across the sampling sites

**Fig. S6** Modified Gibbs diagram illustrating the geochemical processes controlling water quality

**Fig. S7** Water Quality Indices (WQIs) for groundwater and wetland water
