# Supplementary material for: Geochemical assessment of soil and water around abandoned gold mine tailings dams: evidence from the Welkom Goldfield, South Africa
Source: Environ Geochem Health. 2026 Oct 4;48(15):618. doi: 10.1007/s10653-026-03513-0 (PMC13633954; doi:10.1007/s10653-026-03513-0)
Supplement: Supplementary file 2 [file 10653_2026_3513_MOESM2_ESM.docx]

**Table S1** Percentage recoveries, CRMs, R^2^, LODs and LOQs for analysed PTEs in soil samples

| **a** | **Al** | **As** | **Ba** | **Co** | **Cr** | **Cu** | **Fe** | **Mn** | **Ni** | **Pb** | **Ti** | **U** | **V** | **Zn** |
| --- | --- | --- | --- | --- | --- | --- | --- | --- | --- | --- | --- | --- | --- | --- |
| %R | 96 | 102 | 101 | 99 | 98 | 97 | 99 | 96 | 97 | 98 | 99 | 90 | 100 | 116 |
| R^2^ | 0.9997 | 0.9998 | 0.9997 | 0.9999 | 0.9999 | 0.9998 | 0.9999 | 0.9996 | 0.9994 | 0.9997 | 0.9997 | 0.9997 | 0.999 | 0.9974 |
| LOD (mg/kg) | 0.02 | 0.0001 | 0.025 | 0.0002 | 0.001 | 0.013 | 0.018 | 0.005 | 0.003 | 0.000 | 0.01 | 0.000 | 0.025 | 0.007 |
| LOQ (mg/kg) | 0.03 | 0.000 | 0.075 | 0.006 | 0.004 | 0.031 | 0.27 | 0.011 | 0.008 | 0.000 | 0.03 | 0.000 | 0.075 | 0.021 |
| **b**  CRM | Ultraspec Multi element Aqueous CRM/ CRM LOT#Water011-02025us | | | | | | | | | | | | | |

%R: percentage recovery; R^2^: coefficient of determination; LOD: limit of detection; LOQ: limit of quantification; CRM: certified reference materials

**Table S2** Percentage recoveries, CRMs, R^2^ , LODs and LOQs for analysed PTEs in water samples

| **a** | Ca | Na | K | Mg | Al | As | B | Co | Cu | Fe | Li | Mn | Ni | Si | U | Zn |
| --- | --- | --- | --- | --- | --- | --- | --- | --- | --- | --- | --- | --- | --- | --- | --- | --- |
| %R | 96 | 96 | 97 | 93 | 96 | 102 | 99 | 99 | 97 | 99 | 98 | 96 | 97 | 100 | 90 | 116 |
| R^2^ | 0.9999 | 0.9998 | 0.9998 | 0.999 | 0.999 | 0.9998 | 0.9999 | 0.999 | 0.999 | 0.9998 | 0.9996 | 0.9999 | 0.9998 | 0.998 | 0.9997 | 0.9974 |
| LOD (mg/L) | 0.005 | 0.001 | 0.003 | 0.027 | 0.02 | 0.0001 | 0.025 | 0.0002 | 0.013 | 0.018 | 0.001 | 0.05 | 0.003 | 0.2 | 0.000 | 0.007 |
| LOQ (mg/L) | 0.16 | 0.004 | 0.008 | 0.078 | 0.03 | 0.000 | 0.075 | 0.006 | 0.031 | 0.027 | 0.025 | 0.011 | 0.008 | 0.010 | 0.000 | 0.021 |
| **b** |  |  |  |  |  |  |  |  |  |  |  |  |  |  |  |  |
| CRM | Ultraspec Multi element Aqueous CRM/ CRM LOT#Water011-02025us | | | | | | | | | | | | | | | |
| **c** | Cl^-^ | SO_4_^2-^ | F^-^ |  |  |  |  |  |  |  |  |  |  |  |  |  |
| %R | 95 | 96 | 94 |  |  |  |  |  |  |  |  |  |  |  |  |  |
| R^2^ | 0.9992 | 0.9999 | 0.9997 |  |  |  |  |  |  |  |  |  |  |  |  |  |
| LOD (mg/L) | 0.66 | 0.66 | 0.04 |  |  |  |  |  |  |  |  |  |  |  |  |  |
| LOQ (mg/L) | 2 | 2 | 0.09 |  |  |  |  |  |  |  |  |  |  |  |  |  |
| **d** |  |  |  |  |  |  |  |  |  |  |  |  |  |  |  |  |
| CRM | Sodium chloride reagent  ≥ 99% | Sodium sulphate  AM1652849 | Sodium  Acetate  Trihydrate  40387 |  |  |  |  |  |  |  |  |  |  |  |  |  |

%R: percentage recovery; R^2^: coefficient of determination; LOD: limit of detection; LOQ: limit of quantification; CRM: certified reference materials

**Table S3** Soil screening values (SSV’s) for metals concentrations (mg/kg) for various land use purposes

| Chemical element | Water Resource Land Uses protection (SSV1) | Informal Residential (SSV2) | Commercial Industrial (SSV2) |
| --- | --- | --- | --- |
| As | 5.8 | 23 | 150 |
| Co | 300 | 300 | 5000 |
| Cr | 6.5 | 6 | 40 |
| Cu | 16 | 1100 | 19000 |
| Mn | 740 | 740 | 12000 |
| Ni | 91 | 620 | 10000 |
| Pb | 20 | 110 | 1900 |
| V | 150 | 150 | 2600 |
| Zn | 240 | 9200 | 150000 |

**Table S4** Background concentrations used to calculate geoaccumulation index and contamination factors (After Kabata-Pendias & Pendias (2001) in Herselman (2007)

| **Chemical element** | **Background values in mg/kg (after Kabata-pendias & Pendias (2001)** |
| --- | --- |
| **Al** | 82300 |
| **As** | 4.8 |
| **Ba** | 628 |
| **Co** | 17 |
| **Cr** | 83 |
| **Cu** | 27 |
| **Fe** | 40900 |
| **Mn** | 950 |
| **Ni** | 44 |
| **Pb** | 17 |
| **Ti** | 4100 |
| **U** | 2.7 |
| **V** | 107 |
| **Zn** | 120 |

**Table S5** Description of the water quality index and the respective categories used to evaluate water quality

| Statistical index | Equation | Description | Categories | References |
| --- | --- | --- | --- | --- |
| Water Quality Index (WQI) | *WQI*= $\sum_{i=1}^{n} SI$ | ***n*:** number of parameters  ***SI*:** sub-index of the *I*^th^ parameter | **WQI<50**: excellent quality  **50-100**: good water quality  **100-200**: poor water quality  **200-300**: very poor water quality  **WQI>** 300: very bad water quality | Rakotondrabe et al., (2017); Chidiac et al., (2023) |

**Table S6** Relative weights assigned for parameters used to determine WQI and the WHO (2011 & 2022) and USEPA* limits

| Parameter | Unit | WHO and *USEPA limit | Weight (*wi*) | Relative weight (*W*i) |
| --- | --- | --- | --- | --- |
| pH | pH units | ≥5.9 and ≤ 7.4 | 3 | 0.090 |
| TDS | mg/L | 500 | 2 | 0.060 |
| Na | mg/L | 200 | 2 | 0.060 |
| Ca | mg/L | 200 | 2 | 0.060 |
| SO_4_ ^2-^ | mg/L | 200 | 4 | 0.121 |
| Cl^-^ | mg/L | 5 | 3 | 0.090 |
| Mn | mg/L | 0.4 | 4 | 0.121 |
| Fe | mg/L | 0.3 | 3 | 0.090 |
| Li | mg/L | 0.06* | 2 | 0.060 |
| B | mg/L | 2.4 | 2 | 0.060 |
| P | mg/L | 1 | 3 | 0.090 |
| Al | mg/L | 0.2 | 3 | 0.090 |
|  |  |  | ***Σwi= 33*** | ***Σ*wi= 1.20** |

**Table S7** Physicochemical parameters and chemical element concentrations (mg/kg) for the soil at the different sampling sites, Ec in µS/cm and detection limits for the chemical elements

|  |  | Sampling Sites | | | | | | | | | | | | | | |
| --- | --- | --- | --- | --- | --- | --- | --- | --- | --- | --- | --- | --- | --- | --- | --- | --- |
|  | **A 1** | **A2** | **B 1** | **B 2** | **C 1** | **C 2** | **D 1** | **D 2** | **TF1** | **TF2** | **Res 1** | **Res 2** | **RBK** | **FM ODEN** | **STC FM** | **VIRG** |
| pH | 4.5 | 5.2 | 6.8 | 5.3 | 5.2 | 4.8 | 5.8 | 7.0 | 3.7 | 3.7 | 7.0 | 7.2 | 7 | 7.2 | 7.2 | 5.3 |
| EC | 58 | 44 | 35 | 29 | 37 | 59 | 105 | 63 | 75 | 75 | 28 | 42 | 56 | 75 | 116 | 8700 |
| Al | 8800 | 6400 | 36000 | 18400 | 12000 | 9200 | 22800 | 24600 | 11200 | 11200 | 14000 | 6800 | 23200 | 26400 | 24800 | 32800 |
| As | 5 | 3 | 6.3 | 4 | 6 | 5.9 | 10 | 17 | 89 | 89 | 4 | 8.0 | 8 | 3 | 6 | 304 |
| Ba | 21 | <10 | 3 | 1 | 87 | 65 | 188 | 72 | <10 | <10 | <10 | <10 | 95 | 13 | 262 | 159 |
| Co | <10 | <10 | 25 | <10 | 38 | <10 | 35 | 12 | 12 | 12 | <10 | <10 | <10 | <10 | <10 | 25 |
| Cr | 154 | 168 | 132 | 110 | 157 | 153 | 168 | 303 | 467 | 467 | 139 | 156 | 141 | 164 | 118 | 207 |
| Cu | 17 | 7 | 26 | 15 | 9 | 20 | 20 | 31 | 24 | 24 | 13 | 11 | 26 | 8 | 11 | 91 |
| Fe | 11200 | 9600 | 22400 | 19200 | 12400 | 10000 | 22400 | 20000 | 21600 | 21600 | 16400 | 10400 | 25200 | 13200 | 34000 | 25200 |
| Mn | 70 | 100 | 778 | 223 | 493 | 201 | 1112 | 289 | 93 | 93 | 96 | 138 | 200 | 116 | 228 | 150 |
| Ni | 12 | <10 | 29 | <10 | 64 | <10 | 14 | 31 | 21 | 21 | <10 | <10 | 12 | <10 | <10 | 52 |
| Pb | 77 | 7 | 13 | 16 | 19 | 10 | 25 | 19 | 94 | 94 | 8 | 9 | 12 | 7 | 13 | 289 |
| Ti | 2867 | 2482 | 3137 | 24500 | 3474 | 2909 | 2890 | 2350 | 1674 | 1674 | 2908 | 2229 | 2907 | 2164 | 2073 | 2455 |
| U | 23 | 1.3 | 24 | 1.6 | 33 | 1.1 | 1.9 | 3.6 | 18 | 18 | 1.2 | 1.5 | 0.8 | 0.8 | 2.4 | 49 |
| V | 52 | 48 | 81 | 64 | 51 | 48 | 102 | 66 | 52 | 52 | 62 | 45 | 89 | 62 | 48 | 69 |
| Zn | 35 | 12 | 64 | 13 | 81 | 19 | 29 | 53 | 89 | 89 | 23 | 15 | 24 | 17 | 40 | 105 |

**Table S8** Igeo and CF for the chemical elements at the sampling sites

|  | **Chemical elements** | | | | | | | | | | | | | | | | | | | | | | | | | | | | |
| --- | --- | --- | --- | --- | --- | --- | --- | --- | --- | --- | --- | --- | --- | --- | --- | --- | --- | --- | --- | --- | --- | --- | --- | --- | --- | --- | --- | --- | --- |
|  | **Al** | | **As** | | **Ba** | | **Co** | | **Cr** | | **Cu** | | **Fe** | | **Mn** | | **Ni** | | **Pb** | | **Ti** | | **U** | | **V** | | **Zn** | | |
| **Sampling Sites** | CF | I_geo_ | CF | I_geo_ | CF | I_geo_ | CF | I_geo_ | CF | I_geo_ | CF | I_geo_ | CF | I_geo_ | CF | I_geo_ | CF | I_geo_ | CF | I_geo_ | CF | I_geo_ | CF | I_geo_ | CF | I_geo_ | CF |  | I_geo_ |
| **Site A** | 0.09 | 0.0 | 0.8 | 0.1 | 0.03 | 0.0 | n.d | n.d | **2** | 0.4 | 0.4 | 0.0 | 0.2 | 0.0 | 0.0 | 0.0 | 0.2 | 0.0 | **2.5** | 0.5 | 0.6 | 0.1 | **4** | 1 | 0.4 | 0.0 | 0.2 |  | 0.0 |
| **Site B** | 0.3 | 0.0 | 1.0 | 0.2 | 0.003 | 0.0 | 1.4 | 0.3 | 1.5 | 0.3 | 0.7 | 0.2 | 0.5 | 0.1 | 0.5 | 0.0 | 0.7 | 0.1 | 0.8 | 0.2 | 0.8 | 0.6 | **4** | 1 | 0.6 | 0.1 | 0.3 |  | 0.0 |
| **Site C** | 0.1 | 0.0 | 1.2 | 0.2 | 0.2 | 0.0 | **2.2** | 0.4 | 0.4 | **2** | 0.5 | 0.1 | 0.2 | 0.0 | 0.4 | 0.0 | **2** | 0.3 | 0.8 | 0.2 | 0.7 | 0.1 | **6** | **1.3** | 0.4 | 0.1 | 0.8 |  | 0.0 |
| **Site D** | 0.2 | 0.0 | **3** | 0.5 | 0.2 | 0.0 | 1.4 | 0.2 | **3** | 0.5 | 1 | 0.2 | 0.5 | 0.1 | 0.7 | 0.0 | 0.5 | 0.1 | 1.2 | 0.3 | 0.6 | 0.1 | 1 | 0.2 | 0.7 | 0.2 | 0.7 |  | 0.0 |
| **TF** | 0.1 | 0.0 | **10** | **2** | 0.0 | 0.0 | 0.7 | 0.1 | **4** | 0.7 | 0.6 | 0.1 | 0.3 | 0.0 | 0.1 | 0.0 | 0.4 | 0.0 | **3** | 0.6 | 0.4 | 0.0 | **4** | 0.7 | 0.4 | 0.0 | 0.9 |  | 0.0 |
| **Res 1** | 0.1 | 0.0 | 0.8 | 0.2 | n.d | n.d | n.d | n.d | 1.6 | 0.3 | 0.5 | 0.0 | 0.4 | 0.0 | 0.1 | 0.0 | n.d | n.d | n.d | n.d | 0.7 | 0.1 | 0.4 | 0.08 | 0.6 | 0.1 | 0.2 |  | 0.0 |
| **Res 2** | 0.1 | 0.0 | 1.6 | 0.3 | n.d | n.d | n.d | n.d | **2** | 0.4 | 0.4 | 0.0 | 0.2 | 0.0 | 0.1 | 0.0 | n.d | n.d | n.d | n.d | 0.5 | 0.1 | 0.2 | 0.1 | 0.4 | 0.0 | 0.1 |  | 0.0 |
| **RBK** | 0.3 | 0.0 | 1.6 | 0.3 | 0.2 | 0.0 | n.d | n.d | **2** | 0.3 | 1 | 0.2 | 0.6 | 0.1 | 0.2 | 0.0 | 0.0 | 0.2 | 0.7 | 0.1 | 0.7 | 0.1 | 0.3 | 0.05 | 0.8 | 0.1 | 0.2 |  | 0.0 |
| **FMODEN** | 0.3 | 0.0 | 0.6 | 0.1 | 0.02 | 0.0 | n.d | n.d | **2** | 0.4 | 0.3 | 0.0 | 0.3 | 0.0 | 0.1 | 0.0 | n.d | n.d | 0.4 | 0.0 | 0.5 | 0.1 | 0.3 | 0.05 | 0.4 | 0.0 | 0.1 |  | 0.0 |
| **STCFM** | 0.3 | 0.0 | 1.2 | 0.2 | 0.4 | 0.0 | n.d | n.d | 1.4 | 0.3 | 0.4 | 0.0 | 0.8 | 0.1 | 0.2 | 0.0 | n.d | n.d | 0.3 | 0.2 | 0.5 | 0.1 | 0.8 | 0.1 | 0.4 | 0.0 | 0.3 |  | 0.0 |
| **VIRG** | 0.4 | 0.0 | **63** | **12** | 0.3 | 0.0 | 1.4 | 0.2 | **2.5** | 0.5 | **3** | 0.7 | 0.6 | 0.1 | 0.2 | 0.0 | **1.2** | 0.2 | **17** | **3** | 0.6 | 0.1 | **18** | **3.6** | 0.6 | 0.1 | 0.8 |  | 0.2 |
| **C*f* classes:** C*f* <1: low contamination; 1 ≤ C*f* < 3: moderate contamination; 3 ≤ C*f* < 6: high contamination; C*f* ≥ 6: very high contamination  **I*geo* classes:** Igeo≤0: background concentration/ practically unpolluted; 0<Igeo≤1: unpolluted to moderately polluted;1<Igeo≤2: moderately polluted; 2<Igeo≤3: moderately to strongly polluted; 3<Igeo≤4: strongly polluted; 4<Igeo≤5: strongly to very strong; Igeo>5: very strong pollution | | | | | | | | | | | | | | | | | | | | | | | | | | | | | |

*n.d* Element below instrument detection limit in sample, Igeo and Cf values indicate increased level of enrichment and very high contamination.*

**Table S9** Assessment scores for the different statistical index indicators for pollution at each sampling site.

| Sampling site | PLI | Risk classification | PERI | Risk classification |
| --- | --- | --- | --- | --- |
| Site A | 0.6 | Uncontaminated | 66 | Low risk |
| Site B | 1.0 | Moderate | 67 | Low risk |
| Site C | 1.2 | Moderate | 70 | Low risk |
| Site D | 1.5 | moderate | 106 | Moderate risk |
| TF | 1.0 | Moderate | 254 | High risk |
| Res 1 | 0.3 | Uncontaminated | 16 | Low risk |
| Res 2 | 0.3 | Uncontaminated | 26 | Low risk |
| RBK | 0.5 | Uncontaminated | 26 | Low risk |
| FMODEN | 0.3 | Uncontaminated | 14 | Low risk |
| STCFM | 0.5 | Uncontaminated | 20 | Low risk |
| VIRG | 1.6 | Moderate | 765 | Very high risk |

**Table S10** Difference between chemical element concentrations and the maximum permissible metal concentration in South African soils for various land use purposes

| Sampling sites | | | | | | | | | | |  |
| --- | --- | --- | --- | --- | --- | --- | --- | --- | --- | --- | --- |
| Metals | **Site A** | **Site B** | **Site C** | **Site D** | **TF** | **Res 1** | **Res 2** | **RBK** | **FMODEN** | **STCFM** | **VIRG** |
| Water resource land uses protective (SSV1) | | | | | | | | | | | |
| As | -1.8 | -0.65 | **0.15** | **7.7** | **40.7** | -1.8 | **2.2** | **2.2** | -2.8 | 0.2 | **298.2** |
| Co | n.d | -283 | -276 | -277 | -289 | n.d | n.d | n.d | n.d | n.d | -275 |
| Cr | 154.5 | **114.5** | **148.5** | **229** | **289.5** | **132.5** | **149.5** | **134.5** | **157.5** | **11.5** | **200.5** |
| Cu | -4 | -0.5 | -1.5 | **9.5** | 0 | -3 | -5 | **10** | -8 | -5 | **75** |
| Ni | -85 | -76.5 | -59 | -68.5 | -80.5 | n.d | n.d | -71 | n.d | n.d | -39 |
| Pb | **22** | -5.5 | -5.5 | **2** | **21** | n.d | n.d | -8 | -13 | -7 | **269** |
| V | -50 | -77.5 | -100 | -66 | -102 | -88 | -105 | -61 | -88 | -102 | -81 |
| Zn | -216 | -201 | -190 | -199 | -183 | -217 | -225 | -216 | -233 | -200 | -135 |
| Informal residential area (SSV2) | | | | | | | | | | | |
| As | -19 | -17 | -17 | -9.5 | **23** | -19 | -15 | -15 | -20 | -17 | **281** |
| Co | n.d | -283 | -276 | -277 | -289 | n.d | n.d | n.d | n.d | n.d | -275 |
| Cr | **155** | **115** | **149** | **229.5** | **289.5** | **133** | **150** | **135** | **158** | **12** | **201** |
| Cu | -1088 | -1079 | -1085 | -1074 | -1084 | -1087 | -1089 | -1074 | -1092 | -1089 | -1009 |
| Ni | -608 | -591 | -556 | -597.5 | -609.5 | n.d | n.d | -608 | n.d | n.d | -568 |
| Pb | -26 | -91 | -81 | -88 | -08 | -102 | -101 | -98 | -103 | -97 | **179** |
| V | -50 | -77.5 | -100 | -66 | -102 | -88 | -105 | -61 | -88 | -102 | -81 |
| Zn | -9176 | -9161 | -9150 | -9159 | -9143 | -9177 | -9185 | -9176 | -9183 | -9160 | -9095 |
| Commercial industrial (SSV2) | | | | | | | | | | | |
| As | -146 | -145 | -144 | -136.5 | -103.5 | -144 | -142 | -142 | -147 | -144 | **154** |
| Co | n.d | -4988 | -4962 | -4976 | -499.4 | n.d | n.d | n.d | n.d | n.d | -4975 |
| Cr | **121** | **81** | **115** | **195.5** | **256** | **99** | **116** | **101** | **124** | **195.5** | **167** |
| Cu | -18988 | -18979 | -1898 | -18975 | -1894 | -1898 | -1897 | -18992 | -18974 | -18989 | -18909 |
| Ni | -9994 | -9985 | -9968 | -9977 | -18989 | n.d | n.d | -9988 | n.d | n.d | -9948 |
| Pb | -1858 | -1885 | -1881 | -1833 | -1849 | -1892 | -1891 | -1888 | -1893 | -1887 | -1611 |
| V | -2550 | -2527 | -2550 | -2516 | -2552 | -2538 | -2555 | -2511 | -2538 | -2552 | -2531 |
| Zn | -149976 | -149961 | -149950 | -149969 | -149943 | -149977 | -149989 | -149976 | -149983 | -149969 | -149895 |

| **Analyte** | **TotAs** | **LeachAs** | **TotCo** | **LeachCo** | **TotCr** | **LeachCr** | **TotFe** | **LeachFe** | **TotMn** | **LeachMn** | **TotNi** | **LeachNi** | **TotalPb** | **LeachPb** | **TotU** | **LeachU** | **TotZn** | **LeachZn** |
| --- | --- | --- | --- | --- | --- | --- | --- | --- | --- | --- | --- | --- | --- | --- | --- | --- | --- | --- |
| Tot_As | 1 |  |  |  |  |  |  |  |  |  |  |  |  |  |  |  |  |  |
| Leach_As | ,536* | 1 |  |  |  |  |  |  |  |  |  |  |  |  |  |  |  |  |
| Tot_Co | ,636** | 0,338 | 1 |  |  |  |  |  |  |  |  |  |  |  |  |  |  |  |
| Leach_Co | 0,424 | ,525* | ,569* | 1 |  |  |  |  |  |  |  |  |  |  |  |  |  |  |
| Tot_Cr | 0,483 | 0,481 | 0,494 | 0,448 | 1 |  |  |  |  |  |  |  |  |  |  |  |  |  |
| Leach_Cr | 0,366 | 0,374 | 0,193 | 0,483 | 0,42 | 1 |  |  |  |  |  |  |  |  |  |  |  |  |
| Tot_Fe | ,594* | 0,144 | 0,393 | 0,117 | -0,021 | 0,14 | 1 |  |  |  |  |  |  |  |  |  |  |  |
| Leach_Fe | 0,059 | 0,271 | -0,401 | -0,298 | 0,14 | 0,14 | -0,062 | 1 |  |  |  |  |  |  |  |  |  |  |
| Tot_Mn | 0,305 | -0,094 | ,546* | -0,189 | -0,178 | -0,364 | 0,392 | -0,229 | 1 |  |  |  |  |  |  |  |  |  |
| Leach_Mn | 0,209 | ,615* | 0,34 | ,858** | 0,369 | 0,364 | 0,014 | -0,127 | -0,258 | 1 |  |  |  |  |  |  |  |  |
| Tot_Ni | ,709** | 0,467 | ,885** | ,699** | ,522* | 0,21 | 0,44 | -0,418 | 0,364 | 0,465 | 1 |  |  |  |  |  |  |  |
| Leach_Ni | 0,21 | ,502* | 0,433 | ,758** | 0,269 | 0,389 | 0,041 | -,520* | -0,185 | ,802** | ,523* | 1 |  |  |  |  |  |  |
| Tot_Pb | ,731** | 0,445 | ,650** | ,573* | 0,364 | 0,365 | ,511* | -0,342 | 0,204 | 0,351 | ,756** | ,515* | 1 |  |  |  |  |  |
| Leach_Pb | -0,208 | 0,194 | -0,438 | -0,309 | -0,116 | -0,282 | -0,149 | 0,431 | -0,145 | 0,004 | -0,288 | -0,121 | -0,085 | 1 |  |  |  |  |
| Tot_U | 0,49 | ,590* | ,690** | ,709** | 0,212 | 0,196 | 0,291 | -0,391 | 0,222 | ,574* | ,738** | ,610* | ,746** | -0,175 | 1 |  |  |  |
| Leach_U | 0,271 | ,667** | 0,329 | ,644** | 0,432 | 0,42 | -0,117 | -0,089 | -0,328 | ,687** | 0,493 | ,729** | 0,479 | 0,253 | ,588* | 1 |  |  |
| Tot_Zn | ,703** | ,586* | ,740** | ,775** | 0,331 | 0,364 | ,580* | -0,182 | 0,244 | ,566* | ,843** | 0,485 | ,759** | -0,302 | ,815** | 0,439 | 1 |  |
| Leach_Zn | 0,382 | ,567* | ,559* | ,896** | ,613* | 0,439 | 0,015 | -0,299 | -0,231 | ,766** | ,736** | ,757** | ,528* | -0,203 | ,607* | ,719** | ,675** | 1 |

**Table S11** Correlation coefficients for selected total and leachable concentrations of chemical elements (mg/kg)

**Table S12** Statistical descriptions of physicochemical parameters (pH and EC in mS/m) and concentrations (mg/L) of major ions and potential toxic elements (PTEs) in water

|  | **WHO (2022)** | **National Water Act (1998)** | **Groundwater (n=4)** | | **Wetlands (n=2)** | | **Mine waters (n=3)** | |
| --- | --- | --- | --- | --- | --- | --- | --- | --- |
|  |  |  | **Median (min-max)** | **Mean ± SD** | **Median (min-max)** | **Mean ± SD** | **Median (min-max)** | **Mean ± SD** |
| **pH** | 6.5–8.5 | 5.5–9.5 | 7.4 (7.2–7.9) | 7.4±0.3 | 8.3 (7.5–9.1) | 8.3±1.1 | 3.4 (2.5–7.7) | 4.5±3 |
| **Electrical conductivity (EC)** | 0.4 | 70 | 139 (88–295) | 165±91 | 718 (160–1276) | 718±789 | 621 (327–1102) | 683±391 |
| **Total dissolved solids (TDS)** | 1000 | - | 903 (564–2092) | 1115.5±684 | 4916 (948–8884) | 4916±5612 | 6236 (4058–9352) | 6548±2660 |
| **Dissolved oxygen (DO)** | - | - | 7.6 (7.5–8.0) | 7.7±0.2 | 7.7 (7.7–7.8) | 7.8±0.07 | 7.9 (7.9–7.9) | 8±0 |
| **HCO_3_^-^** | 400 | - | 402 (278–522) | 401±100 | 304 (234–375) | 304±99 | 7.9 (5–229) | 80±129 |
| **Cl^-^** | 250 | - | 144 (82–434) | 201±161 | 2029 (281–3777) | 2029±2471 | 773 (9–2158) | 960.±1095 |
| **SO_4_^2-^** | 200 | - | 174 (95–605) | 262±236 | 715 (195–1235) | 715±735 | 330 (2403–3698) | 3144±667 |
| **Ca** | 200 | - | 119 (49–312) | 150±116 | 61 (40–82) | 61±21 | 487 (128–633) | 416.0±269 |
| **Na** | 200 | - | 147 (47–232) | 144±75 | 1141 (208–2075) | 1141±1320 | 244 (1–1524) | 589±818 |
| **K** | 10 | - | 16.5 (4.6–28) | 16±10 | 82 (20–144) | 82±87 | 6 (0.5–11) | 5.9±5 |
| **Mg** | 150 | - | 40.5 (27–91) | 49±28 | 120 (39–201) | 120±114 | 530 (384–667) | 527±142 |
| **Chemical composition of PTEs** | | | | | | |  |  |
| **Al** | 0.1–0.2 | - | 0.1 (0.100–0.180) | 0.1±0 | 0.16 (0.13–0.19) | 0.1±0.1 | 122 (105–138) | 121.7±17 |
| **As** | 0.01 | 0.02 | - | - | 1.2 (0.01–2) | 1.2±2 | 0.00 (0.007–2) | 0.72±1 |
| **B** | 2.4 | 1 | 0.09 (0.025–0.72) | 0.2±0.3 | 1.3 (0.3–2.3) | 1.3±1.4 | 0.07 (0.04–0.23) | 0.1±0.1 |
| **Co** | 0.1 | - | - | - | - | - | 1.3 (0.9–5.3) | 2.5± 2.45 |
| **Cu** | 2.0 | 0.01 | 0.01 (0.00–0.05) | 0.01±0.03 | - | - | 1.3 (0.9–5.3) | 2.5±2.4 |
| **F** | 1.5 | 1 | 0.4 (0.4–0.6) | 0.45±0.1 | 0.65 (0.6–0.7) | 0.65±0.70 | 0.4 (0.2 -1.0) | 0.5 ±0.41 |
| **Fe** | 2 | 0.3 | 0.09 (0.02–0.3) | 0.1±0.1 | 0.225 (0.09–0.35) | 0.225 ± 0.18 | 404 (2.8–427) | 278.0±239 |
| **Li** | 0.06 | - | 0.06 (0.02–0.12) | 0.07±0.04 | 0.0 | 0.0 | 0.2 (0.13–0.4) | 0.26± 0.17 |
| **Mn** | 0.08 | 0.1 | 0.038 (0.03–7.38) | 1.87±3 | 0.04 (0.0–0.047) | 0.04±0.008 | 4.6 (4.3–42) | 17±2 |
| **Ni** | 0.07 | - | - | - | 0.6 (0.0–1.2) | 0.6±0.8 | 2.5 (0.6–13) | 5.3±6.6 |
| **Si** | 10 | - | (10–17) | 11.5±7.8 | 0.6 (0.6–0.6) | 0.6±0 | 2.6 (2.6–30) | 11±15.8 |
| **U** | 0.03 | - | 0.008 (0.00–0.02) | 0.01±0.01 | - | - | 2.5 (0.9–5.8) | 3.1 ±2.5 |
| **Zn** | 4 | 0.1 | 0.055 (0.0–1.9) | 0.5±0.9 | - | - | 1.9 (0.1–21) | 7.7 ±11.5 |
